# Isolated Facial Nerve Palsy Due to Temporal Bone Metastasis

**DOI:** 10.7759/cureus.26931

**Published:** 2022-07-17

**Authors:** Sangeetha Isaac, Mohammed Afraz Pasha, Yoon Sim Yap, Jason Chan

**Affiliations:** 1 Internal Medicine, North Alabama Medical Center, Florence, USA; 2 Division of Medical Oncology, National Cancer Centre Singapore, Singapore, SGP

**Keywords:** aromatase inhibitor, letrozole, metastatic malignancy, breast cancer management, facial nerve paralysis

## Abstract

Isolated facial nerve palsy resulting from temporal bone metastasis is rare and has been sparsely reported in the literature to be the initial presentation of cancer. The most commonly reported sites of origin of such metastases include the breast, lung, kidney, gastrointestinal tract, larynx, prostate, and thyroid, to name a few. Here, we discuss a patient initially presenting with isolated lower motor neuron facial nerve palsy. The diagnosis was revised to that of breast cancer with metastasis to the temporal bone resulting in facial nerve paralysis following the subsequent clinical presentation.

## Introduction

Isolated facial nerve palsy, clinically represented as Bell’s palsy and commonly highlighted as an acute peripheral facial palsy of unknown clinical etiology, accounts for 72% of facial palsies [1]. Bell’s palsy is an acute facial paralysis affecting the 7th cranial nerve and has a reported incidence of about 25 cases per 100,000 population yearly [2]. The clinical manifestation of Bell's palsy encompasses a sudden onset of unilateral facial paralysis, which lasts for a few hours and typically resolves over a period of six months [3]. However, some people are affected permanently; 5-9% have a recurrence, with the average time span between episodes being 10 years.

While isolated occurrences are quite common, multiple recurrent episodes of unilateral facial paralysis are rare. Meanwhile, omission of congenital abnormalities, systemic inflammation or infection, or trauma as a prime cause of facial palsy should raise a suspicion of metastatic tumors to the temporal bone, as well as primary malignancies. Temporal bone metastasis is a rare clinical event and imposes a diagnostic challenge due to its asymptomatic feature in most cases [4]. The mounting clinical report displays the involvement of temporal bone metastasis as the cause of facial nerve palsy [5-7]. Here, we report an unusual case where the initial presentation of lower motor neuron facial nerve palsy was presumptively managed as Bell’s palsy and was diagnosed to have metastatic breast cancer following subsequent clinical manifestation.

## Case presentation

A 54-year-old Malay lady presented to the emergency department with the inability to close her left eye, with an associated hemifacial droop of one week's duration. Examination findings were keeping in with idiopathic facial nerve palsy with lower motor neuron lesion. The rest of her general and neurological examination was unremarkable. A presumptive diagnosis of Bell’s palsy was made and she was discharged on a course of oral prednisone.

She presented three months later with generalized weakness, bony pain, and unexplained weight loss and breast mass. She was hemodynamically stable on presentation. Facial examination revealed persistence of left facial nerve paralysis. Neurological examination was otherwise unremarkable. She was noted to have a 5 cm fungating right breast mass with an ulcerated surface, which was a new finding.

**Figure 1:**
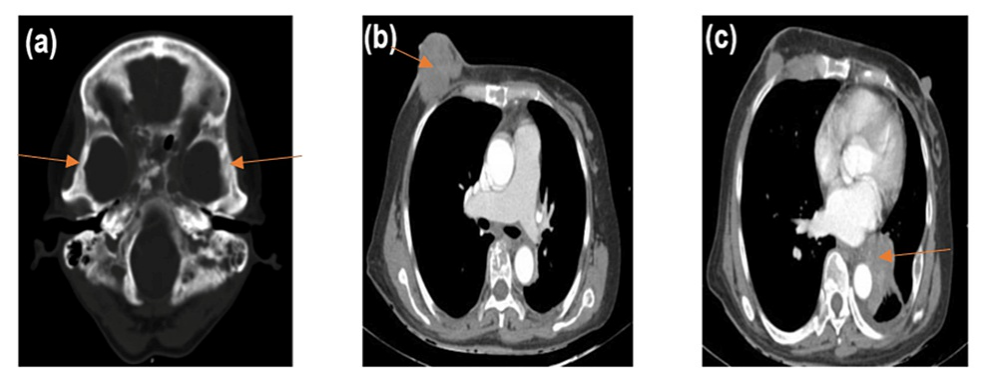
CT images acquired in the axial plane demonstrating (A) temporal bony metastases, (B) a fungating right breast mass, and (C) lung metastases.

**Figure 2:**
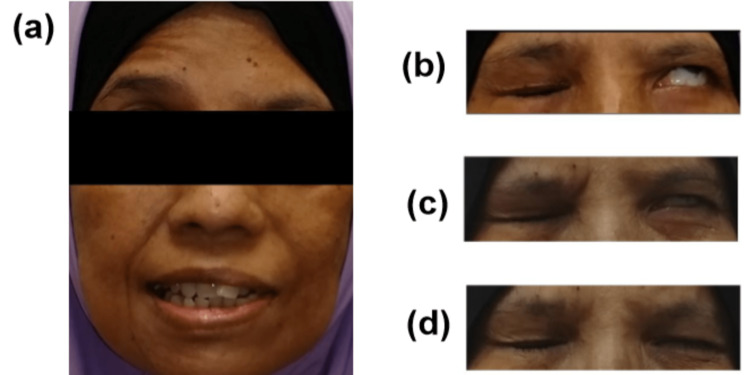
Clinical images demonstrating (A) left hemifacial palsy with associated (B) Bell's phenomenon at diagnosis. (C) Clinical improvement at six weeks into treatment and (D) at six months into treatment.

Histopathology of the breast lesion confirmed invasive ductal carcinoma, with positive immunostaining for estrogen and progesterone receptors, as well as negativity for Cerb-B2. Further workup revealed extensive systemic metastases including deposits at bilateral temporal petrous apices consistent with bony metastasis (Figure 1). The patient was started on letrozole, a non-steroidal aromatase inhibitor. Subsequent evaluation after six weeks of treatment revealed improvement in energy levels and pain scores, along with an interval improvement of the facial palsy (Figure 2).

## Discussion

The prevalent clinical cause of unilateral facial paralysis is Bell’s palsy, Ramsay Hunt syndrome, Lyme disease, and complications of chronic otitis [8]. However, isolated facial nerve palsy as the first sign of temporal bone metastasis is a rare clinical scenario. Metastatic spreads of tumors to the temporal bone are scarce and generally originate from the breast, lung, or kidney via hematogenous spread [9-11]. Other pathways of dissemination are possibly meningeal, perineural, and lymphatic. In many cases, the exact prevalence of temporal bone metastasis is not known, since the histological evaluation of the temporal bone is not a routine procedure during autopsy examination of patients with primary malignant cancer.

The most common symptoms reported in patients with temporal bone cancer are hearing loss, facial paralysis, peri-auricular swelling, otorrhea, otalgia, vertigo, aural mass, and tinnitus [12,13]. However, these conditions are not pathognomonic of metastatic temporal bone cancer but are the most frequent symptoms of mastoid infection.

In a study conducted by Maddox, the incidence of facial paralysis in the presence of temporal bone metastases has been reported to be 34%, compared to 50% and 14.9% in the studies of Schuknecht et al. and Gloria-Cruz et al., respectively [11-13]. Thus, the combination of otalgia, facial palsy, and the appearance of peri-auricular swelling as a triad may be suggestive of metastasis to the temporal bone.

Furthermore, inflammatory diseases, systemic infections, or some benign tumors should be considered in the differential diagnoses. A case reported by Suryanarayanan et al. displayed a secondary deposit in the temporal bone mimicking facial nerve schwannoma and stressed the importance of having a suspicion for metastatic tumors in a patient with a previous history of malignancy [14-16].

## Conclusions

In conclusion, metastatic tumors to the temporal bone are rare, highlighting the need for the physician to consider them in the differential diagnoses of patients presenting with facial nerve palsy. The treatment of metastatic temporal bone lesions may involve systemic chemotherapy with local radiotherapy to the affected temporal region. In the present case, the patient was treated with letrozole, and reevaluation after six weeks revealed improvement in energy levels and pain scores, along with an interval improvement of the facial palsy.

## References

[1] Gilden DH (2004). Clinical practice. Bell's palsy. N Engl J Med.

[2] Nivetha SK (2016). Bell palsy’s and its clinical significance - a review. J Pharm Sci Res.

[3] Katusic SK, Beard CM, Wiederholt WC, Bergstralh EJ, Kurland LT (1986). Incidence, clinical features, and prognosis in Bell's palsy, Rochester, Minnesota, 1968-1982. Ann Neurol.

[4] Djeric D, Boricic I, Tomanovic N, Cvorovic L, Blazic S, Folic M, Djoric I (2017). The facial palsy as first symptom of the temporal bone lung cancer metastasis. Braz J Otorhinolaryngol.

[5] Lan MY, Shiao AS, Li WY (2004). Facial paralysis caused by metastasis of breast carcinoma to the temporal bone. J Chin Med Assoc.

[6] Choi SH, Park IS, Kim YB, Hong SM (2014). Unusual presentation of a metastatic tumor to the temporal bone: severe otalgia and facial paralysis. Korean J Audiol.

[7] Yildiz O, Buyuktas D, Ekiz E, Selcukbiricik F, Papila I, Papila C (2011). Facial nerve palsy: an unusual presenting feature of small cell lung cancer. Case Rep Oncol.

[8] Patel DK, Levin KH (2015). Bell palsy: clinical examination and management. Cleve Clin J Med.

[9] Streitmann MJ, Sismanis A (1996). Metastatic carcinoma of the temporal bone. Am J Otol.

[10] Cumberworth VL, Friedmann I, Glover GW (1994). Late metastasis of breast carcinoma to the external auditory canal. J Laryngol Otol.

[11] Schuknecht HF, Allam AF, Murakami Y (1968). Pathology of secondary malignant tumors of the temporal bone. Ann Otol Rhinol Laryngol.

[12] Gloria-Cruz TI, Schachern PA, Paparella MM, Adams GL, Fulton SE (2000). Metastases to temporal bones from primary nonsystemic malignant neoplasms. Arch Otolaryngol Head Neck Surg.

[13] Maddox HE 3rd (1967). Metastatic tumors of the temporal bone. Ann Otol Rhinol Laryngol.

[14] Suryanarayanan R, Dezso A, Ramsden RT, Gillespie JE (2005). Metastatic carcinoma mimicking a facial nerve schwannoma: the role of computerized tomography in diagnosis. J Laryngol Otol.

[15] Jones HM (1969). Case of metastasis in the temporal bone from a carcinoma of the breast. J Laryngol Otol.

[16] Isaac SP, Chan JY, Yap YS (2017). Isolated facial nerve palsy secondary to temporal bone metastasis. (P5.182). Neurology.

